# Identification of an immunological signature of long COVID syndrome

**DOI:** 10.3389/fimmu.2024.1502937

**Published:** 2025-01-08

**Authors:** Gisella Guerrera, Manolo Sambucci, Eleonora Timperi, Mario Picozza, Andrea Misiti, Roberta Placido, Silvia Corbisiero, Silvia D’Orso, Andrea Termine, Carlo Fabrizio, Francesca Gargano, Sharon Eleuteri, Luisa Marchioni, Veronica Bordoni, Luigi Coppola, Marco Iannetta, Chiara Agrati, Giovanna Borsellino, Luca Battistini

**Affiliations:** ^1^ Neuroimmunology Unit, Santa Lucia Foundation IRCCS, Rome, Italy; ^2^ Data Science Unit, Santa Lucia Foundation IRCCS, Rome, Italy; ^3^ UOS Terapia Intensiva Postoperatoria e Assistenza Subintensiva, National Institute for Infectious Diseases IRCCS Lazzaro Spallanzani, Rome, Italy; ^4^ Unit of Pathogen specific Immunity, Research Area of Hematology and Oncology, Bambino Gesù Children’s Hospital IRCCS, Rome, Italy; ^5^ Infectious disease Clinic, Policlinico Tor Vergata of Rome, Rome, Italy; ^6^ Department of Systems Medicine, Infectious Disease Clinic, Tor Vergata University, Rome, Italy

**Keywords:** SARS-CoV-2 infection, Long COVID, immune response, immunological signature, post-acute sequelae of COVID19, immune dysregulation

## Abstract

**Introduction:**

Acute COVID-19 infection causes significant alterations in the innate and adaptive immune systems. While most individuals recover naturally, some develop long COVID (LC) syndrome, marked by persistent or new symptoms weeks to months after SARS-CoV-2 infection. Despite its prevalence, there are no clinical tests to distinguish LC patients from those fully recovered. Understanding the immunological basis of LC is essential for improving diagnostic and treatment approaches.

**Methods:**

We performed deep immunophenotyping and functional assays to examine the immunological profiles of LC patients, individuals with active COVID-19, recovered patients, and healthy donors. This analysis assessed both innate and adaptive immune features, identifying potential biomarkers for LC syndrome. A Binomial Generalized Linear Model (BGLM) was used to pinpoint immune features characterizing LC.

**Results:**

COVID-19 patients exhibited depletion of innate immune cell subsets, including plasmacytoid and conventional dendritic cells, classical, non-classical, and intermediate monocytes, and monocyte-derived inflammatory dendritic cells. Elevated basal inflammation was observed in COVID-19 patients compared to LC patients, whose immune profiles were closer to those of healthy donors and recovered individuals. However, LC patients displayed persistent immune alterations, including reduced T cell subsets (CD4, CD8, Tregs) and switched memory B cells, similar to COVID-19 patients. Through BGLM, a unique adaptive immune signature for LC was identified, featuring memory CD8 and gd T cells with low proliferative capacity and diminished expression of activation and homing receptors.

**Discussion:**

The findings highlight a unique immunological signature associated with LC syndrome, characterized by persistent adaptive immune dysregulation. While LC patients displayed recovery in innate immune profiles comparable to healthy and Recovered individuals, deficits in T cell and memory B cell populations were evident, differentiating LC from full recovery. These findings provide insights into LC pathogenesis and may support the development of diagnostic tools and targeted therapies.

## 1 Introduction

The coronavirus pandemic started in 2019 has had extraordinary impacts on every aspect of human life. Infection with SARS-CoV2 sometimes has long-lasting effects, and leaves traces like scars in the immune system. Over one billion human infections have been documented, a likely underestimate given the uneven provenance of the data, since third-world countries did not perform testing consistently. In most cases COVID-19 is a mild disease, and infections can be asymptomatic (1, 2). In a small fraction of individuals, however, SARS-CoV2 infection is a nearly catastrophic event characterized by excessive inflammation and multi-system failure (3, 4). Several factors are associated with the risk of severe disease, such as senescence, immune depression, obesity, male sex, and lung disease, and most of these conditions are associated with very high levels of basal inflammation and/or immune dysregulation (5–7). Innate immune cells are involved in the devastations induced by excessive inflammation. During acute COVID-19, granulocytes, monocytes, and dendritic cells show markers of hyper/dysfunction (8, 9). Current knowledge on the disease states that failure or delay in mounting an initial antiviral response upon infection determines the extent of the tissue damage and magnitude of the immune responses that follow. However, excessive inflammation is often driven or maintained by the dysregulation of minor cell populations rather than by the debacle of the whole immune system. For instance, pDC represent only 0.2-0.8% of peripheral blood mononuclear cells (PBMC) in humans, but their malfunction has severe consequences in antiviral responses (10–13). The adaptive immune system is also dysregulated during acute COVID-19 infection (14, 15). SARS-COV-2 shuffles adaptive immune repertories and COVID-19-induced remodeling of peripheral lymphocytes. Variations in the peripheral T and B cells from the COVID-19 patients revealed a correlation of humoral immune response and T-cell immune memory with disease severity (16). Upon acute COVID-19 infection some patients may display a post-acute LC syndrome described also in children (17), characterized by immunological dysfunctions persisting also up to years following initial mild-to-moderate SARS-CoV-2 infection (18). In LC patients a persisting immune activation possibly due to defective virus clearance and chronic infection has been shown (19–24). Here, we performed deep immunophenotyping including *ex vivo* functional assays to profile the immunological landscape of LC and COVID-19 patients and individuals who had recovered from COVID-19 infection. We also included a cohort of HD. Currently, there is no availability of a clinical test that may define LC syndrome. Therefore, our objective was to pinpoint markers that could help in classifying LC syndrome. In this study, we outlined immunological characteristics, including activation markers of both adaptive and innate immune cells. These markers are intended to be incorporated into clinical tests as valuable tools to facilitate the diagnosis of LC syndrome.

## 2 Materials and methods

### 2.1 Study design and patients’ characteristics

**Figure 1:**
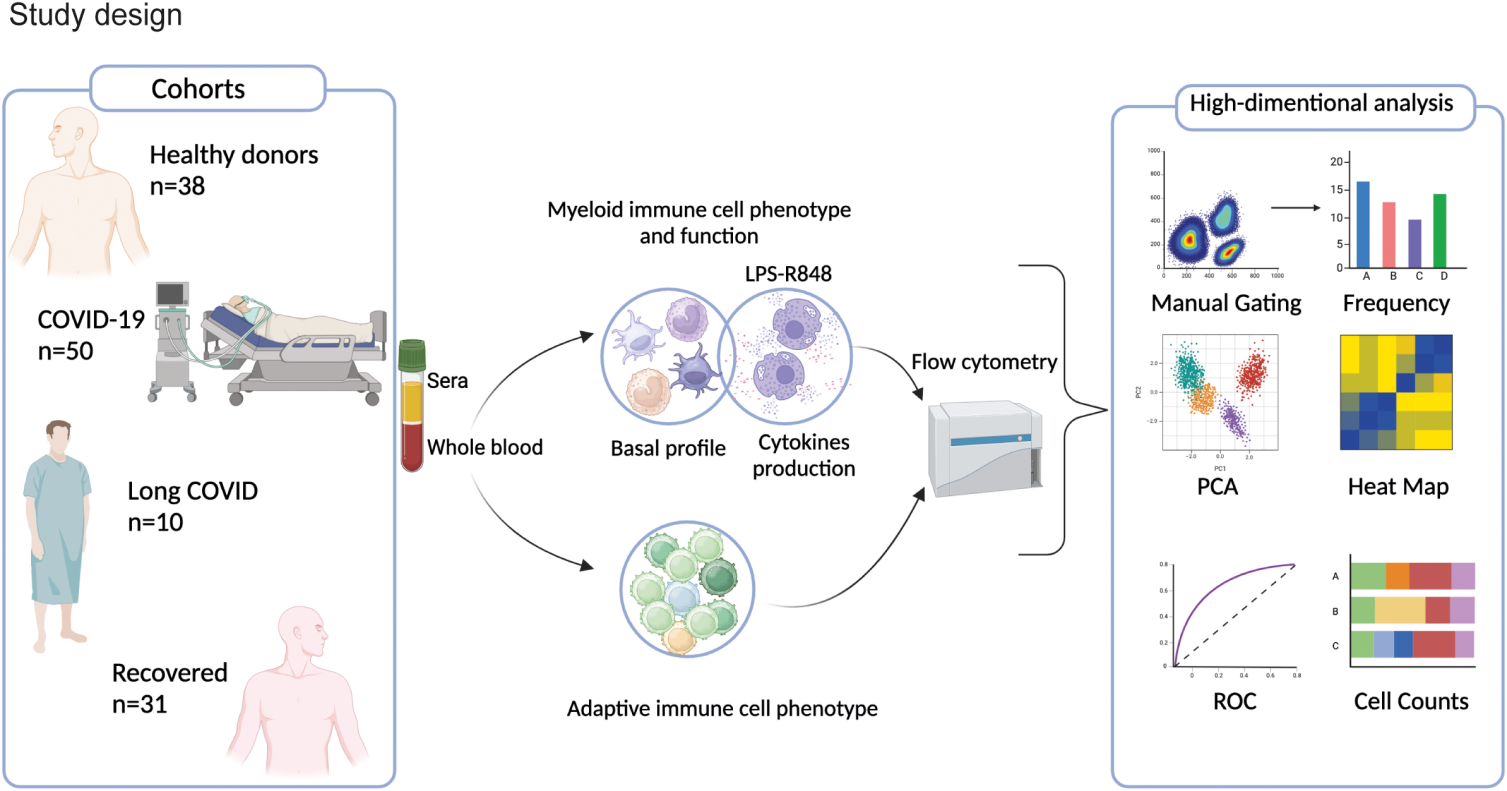
Study design. Cohort of enrolled COVID (N=50) and LC (N=10) patients and HD (N=38) and Recovered (N=31) subjects. Myeloid and lymphoid cell populations, cytokine/chemokine production and activation markers were analyzed by high-parameter flow cytometry and by bioinformatic modeling.

The study includes four participant groups: COVID-19 patients (N=50) (5.8 **±** 3.1 days of sampling post-infection) hospitalized at the National Institute for Infectious Diseases “Lazzaro Spallanzani” IRCCS; Recovered patients (N=31) who successfully overcame COVID-19 and were collected around at 45**±**15 days post-infection; Long COVID patients (N=10) (279**±** 201 days sampling post-infection) experiencing new or persistent symptoms from 3 months post-SARS-CoV-2 infection, selected from the Infectious Diseases Unit of the University Hospital “Tor Vergata” and the University Hospital “Umberto I” of Rome; and Healthy Donors (HD, N=38). Study design and demographic data are provided in **Figure 1**; **Table 1**. Clinical data of COVID and LC are available in **Supplementary Tables 1**, **2**, respectively. All participants signed informed consent forms in accordance with national legislation and the World Medical Association’s Code of Ethics for Medical Research Involving Human Subjects (Declaration of Helsinki). Blood sample collected from peripheral venous blood in heparinized tubes (BD Vacutainer) for immunological studies, was immediately processed as described below.

**Table 1:** Study demographic.

Donor Features	COVID19	Recovered	LC	HD
N	50	31	10	38
Sex (female/male)	17/33	16/15	8/2	13/25
Age (y.o.)	54.8 ± 12.8	46.9 ± 8.8	64.5 ± 8.9	52.4 ± 13.8
Day of sampling after infection	5.8 ± 3.1	45±15	279 ± 201	N/A
COVID Hospitalization (Y/N)	50/0	0/31	10/0	N/A
COVID-vaccination (Y/N)	0/50	0/31	6/4	N/A
Ventilation during hospitalization (Y/N)	35/15	N/A	7/3	N/A
Associated comorbidities (Y/N)	24/26	N/A	9/1	N/A

Demographic information for test subjects with either COVID19 infection (COVID19), subjects recovered without lingering symptoms (Recovered) subjects with lingering symptoms more than 3 months after diagnosed SARS-CoV-2 infection (LC), and subjects without COVID19 infection (HD).

### 2.2 Stimulation of innate immune cells *in vitro*

Freshly collected whole blood was incubated for 3 hours alone or with different stimuli to mimic bacterial and viral stimulation. The stimuli used were lipopolysaccharide (LPS from E. coli, 1µg/ml, Sigma), a TLR4 agonist which is a bacterial endotoxin; and R848 (1µg/ml, Invivogen), a TLR7/8 agonist mimicking single-stranded RNA. Brefeldin-A (10 µg/ml, Sigma) was added as a protein transport inhibitor, to enhance intracellular cytokine staining signals by blocking transport processes during cell activation.

### 2.3 Flow cytometry staining and acquisition

We obtained information on the total leukocyte pool by performing cytometric analyses on cell suspensions obtained by ammonium chloride-based, hypotonic red blood cell lysis of whole blood. Cells, whether stimulated or not, were pelleted in V-bottom 96-well plates and resuspended in 30 µl of antibodies (Abs) mixture diluted in Brillant Stain Buffer (BD Biosciences). All Abs listed in **Supplementary Materials** were incubated with the cells for 15 min at room temperature (RT) and were used at pre-optimized concentrations. After incubation, cells were washed and fixed either in FoxP3 fixation/permeabilization buffer (Thermo Fisher Scientific) for 20 min at RT (for later staining of nuclear proteins) or in 4% formaldehyde in PBS (for intracellular cytokine staining). After fixation, cells were washed and incubated with 30 µl of Abs mixture diluted in either FoxP3 permeabilization buffer or in a 0.5% w/v solution of saponin/PBS. The cell suspensions were then washed and samples acquired on a 6-laser CytoFLEX LX (Beckman Coulter). Absolute counts were obtained on the same day using a volumetric lyse-no-wash flow cytometry method on a separate blood aliquot as previously described (25). Flow cytometry (FC) data were analyzed with FlowJo v.10.7 (BD Biosciences) and CytExpert v2.2.0.97 (Beckman Coulter).

### 2.4 Robust principal component analysis

Principal Components of each dataset (myeloid population counts, baseline counts of cytokine- and chemokine- producing myeloid cells, counts of cytokine- and chemokine- producing myeloid cells after LPS and R848 stimulations, and lymphocyte population counts for all individuals) were calculated using the scikit-learn Python package (26) preceded by Robust Principal Component Analysis (RPCA) (27) utilizing the Linearized Alternating Direction method (28) to reduce effects from outlying observations.

### 2.5 Data pre-processing and feature selection for machine learning training

The following steps were applied for each of the above datasets: to pre-process the data, we removed zero variance predictors (immunological variables with no changes across patients), imputed missing values using a median imputation strategy, applied a logarithmic transformation (base 10) to stabilize variance, and centered and scaled the data to standardize the range of values across different features. Each dataset was then split into a training and a test set with a 60%/40% split. For feature selection, we used the *SelectFdr* function from scikit-learn (26), performing ANOVAs for all variables in each dataset and applying a 0.05 threshold on the adjusted p-value (Benjamini-Hochberg procedure).

### 2.6 Extreme gradient boosting model training and variable importance calculation

We trained an XGBoost (eXtreme Gradient Boosting) (29) model using k-fold cross-validation on each training set and evaluated their performances on the respective test sets using the area under the Receiver Operating Curve (auROC) and overall accuracy metrics, using the One-vs-Rest scheme. In this way each class (COVID, HD, LC, Recovered) is compared against all the others combined. Finally, we assessed variable importance by computing the fractional contribution of each feature to the model based on the total gain of the corresponding feature’s splits (“gain” method) scaled to a 0-100 range. Higher values mean a more important predictive feature. This approach allowed us to understand the contribution of each feature to the model and improve its generalization capabilities.

### 2.7 Local optimum analysis for classification between HD/recovered and long COVID

For each analyzed dataset, after filtering features with statistically significant differences between HD/Recovered and LC based on the Wilcoxon Rank Sum test for independent groups with the Holm p-value correction, we fitted a Binomial Generalized Linear Model (GLM) for each feature to find the local optimum (the best performing predictor in differentiating LC from HD/Recovered) based on the auROC and the accuracy of the GLM model.

### 2.8 Statistical tests

For each dataset (myeloid population counts, baseline counts of cytokine- and chemokine- producing myeloid cells, counts of cytokine- and chemokine- producing myeloid cells after LPS and R848 stimulations, and lymphocyte population counts), we used the Wilcoxon Rank Sum test for independent groups with the Holm p-value correction for the comparison of the resulting top variables’ distributions in each group of subjects (HD, COV, LC, Recovered). For every test the result is deemed statistically significant when adjusted *p*-value < 0.05.

## 3 Results

### 3.1 LC patients exhibited a distribution of myeloid cell populations similar to unexposed HD and recovered subjects

**Figure 2:**
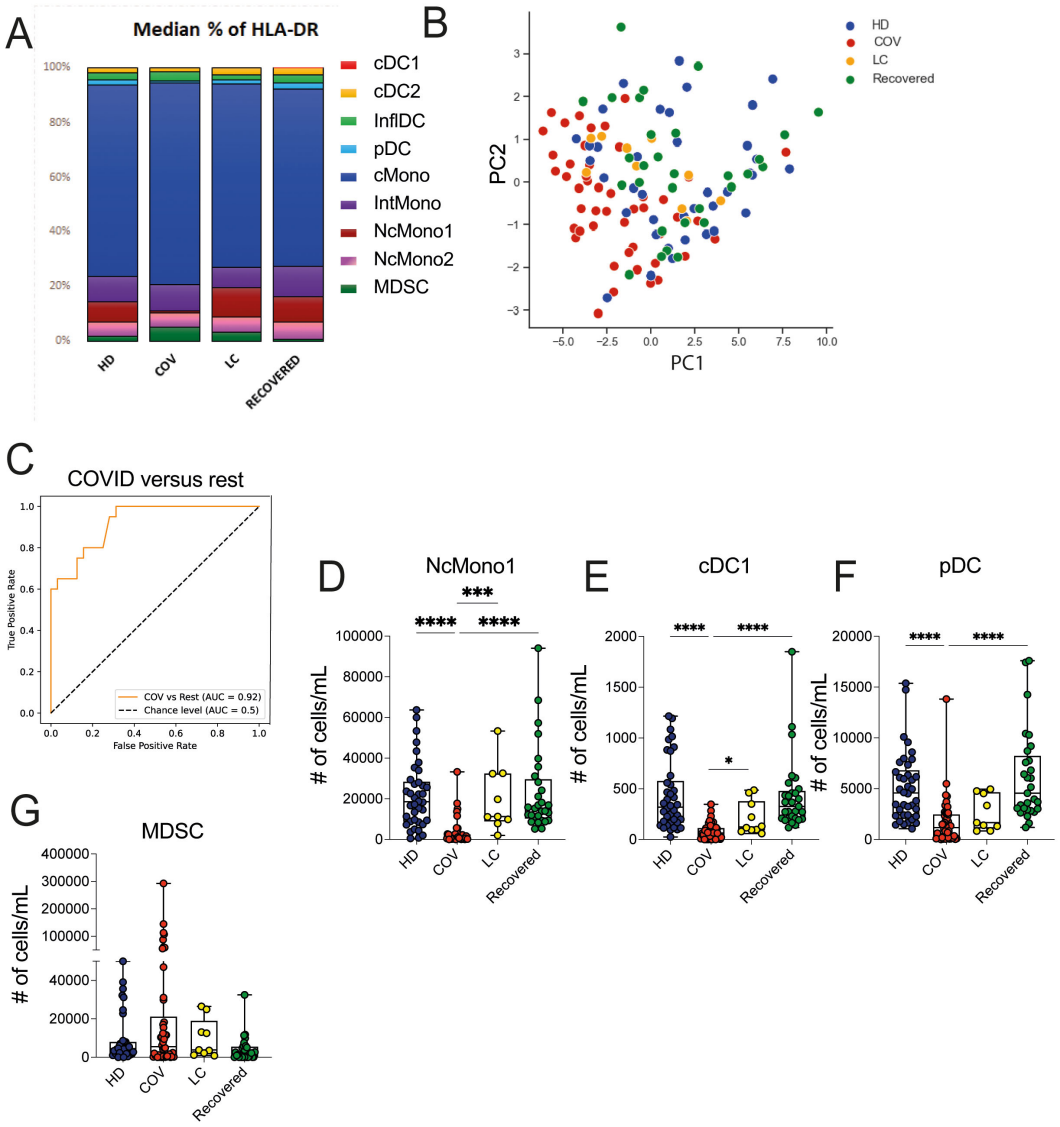
Myeloid cell population counts and distribution in HD, COVID, LC and Recovered groups. **(A)** Graph showing the percentage of DC (pDC, cDC1, cDC2), monocytes (NcMono1, NcMono2, cMono and IntMono) and MSDC cell populations within HLA-DR+ myeloid cells in HD, COVID, LC and Recovered groups based on gating strategy shown in **Supplementary Figure 1A**. **(B)** Principal Component Analysis (PCA) of the myeloid cell subpopulation counts matrix derived from FC data is shown. Each dot represents one variable in HD, COVID, LC and Recovered groups. **(C)** XGBoost model was fitted on each training set and their performances were evaluated on the respective test sets using the auROC metrics. ROC curves of COV vs Rest (including LC, HD, Recovered) auROC value 0.92. **(D–G)** Graph showing the counts obtained by FC data of NcMono1, cDC1, pDC and MDSC cell populations in HD, COVID, LC and Recovered groups. Box and whiskers represent median of values with interquartile range. COVID (N=50), LC (N=10), HD (N=38), Recovered (N=31). Wilcoxon Rank Sum test for independent groups with the Holm *p-*value correction is shown. *** *p*<0.001 **** *p*<0.0001. No symbol, not significant.

With the aim of identifying specific immunological signatures of LC, we examined the immune profile of peripheral blood mononuclear cells (PBMC) through multi parametric FC analysis in individuals hospitalized for COVID-19, individuals with LC, and patients who had successfully recovered from the infection. As a control group, we included unexposed HD (see study design in **Figure 1**). Freshly obtained peripheral blood cells were stained with an antibody panel designed to detect 9 myeloid cell subsets, and using the gating strategy outlined in **Supplementary Figure 1A**. We started out evaluating the frequency of the myeloid immune cell subpopulations, including monocytes, conventional/myeloid dendritic cells 1 and 2 (cDC1 and cDC2), plasmacytoid DCs (pDC), and myeloid-derived suppressing cells (MDSC). Monocytes were further subdivided in classical (cMono: HLADR+CD11c+CD14+CD38+), intermediate (HLA-DR+CD11c+CD14^int^CD38+), non-classical 1 (NcMono1: HLA-DR+CD11c+CD14-CD38-), corresponding to the CD16+ monocyte subset (30), non-classical 2 (NcMono2: HLA-DR+CD11c+CD14^int/low^CD38-), and inflammatory dendritic cells (inflDC: HLA-DR+CD11c+CD14^int/low^CD38+). As expected, classical monocytes (cMono) represented the largest fraction of the myeloid compartment, particularly in the COVID-19 group, which correspondingly showed a significant reduction in the percentage of cDC1, cDC2, pDC, and particularly of non-classical monocytes (NcMono1 and NcMono2) compared to HD, LC, and Recovered individuals (**Figure 2A**). Intriguingly, LC patients displayed a myeloid cell distribution pattern similar to that observed in HD and Recovered groups. In order to give context to the abundance of the different cell populations, and to understand the overall magnitude of the immune response, we calculated absolute cell counts of each myeloid cell population, as previously described (25). Analysis of absolute cell counts of myeloid cell populations through Principal Component Analysis (PCA) revealed the partial clustering of the LC group with HD and Recovered patients, while a slight segregation of the COVID cohort from HD and Recovered patients was observed (**Figure 2B**). Then, in search for immunological features (or combinations thereof) specific to the disease status, we trained an XGBoost classification model utilizing myeloid cell population counts as features and disease status as the target variable. The model’s performance, assessed by the area under the One-vs-Rest receiver operating characteristic curve (auROC), yielded values of 0.92, when classifying the COVID cohort against the other classes (HD, LC, Recovered) combined (**Figure 2C**), and lower values for the HD, LC, and Recovered cohorts (not shown). Through variable importance analysis, the key variables distinguishing the four groups were identified, including NcMono1, cDC1, pDC, IntMono, and cMono myeloid cell populations (**Figures 2D–G** and **Supplementary Figure 1B**). Individual graphs for NcMono1, cDC1, and pDC (**Figures 2D-F**) exhibited consistently lower counts in the COVID group compared to LC and HD Recovered subjects. While monocytes (IntMono, cMono, NcMono2) and DC cell populations (cDC2.2, cDC2, inflDC) demonstrated a general decrease in count in the COVID group, MDSC counts tend to be higher in COVID compared to other groups (**Figure 2G**), as previously reported (31). Individuals experiencing long-lasting symptoms did not exhibit a significant reduction in monocytes and DC cells, as observed during the acute phase of COVID infection. This suggests that COVID-19 patients in the acute phase show a distinctive immunological profile with a specific peripheral deprivation of the myeloid cell subsets, not persisting in individuals with LC.

### 3.2 Myeloid cells from COVID-19 patients show high levels of basal cytokine and chemokine production, and return to normal basal levels in LC patients

**Figure 3:**
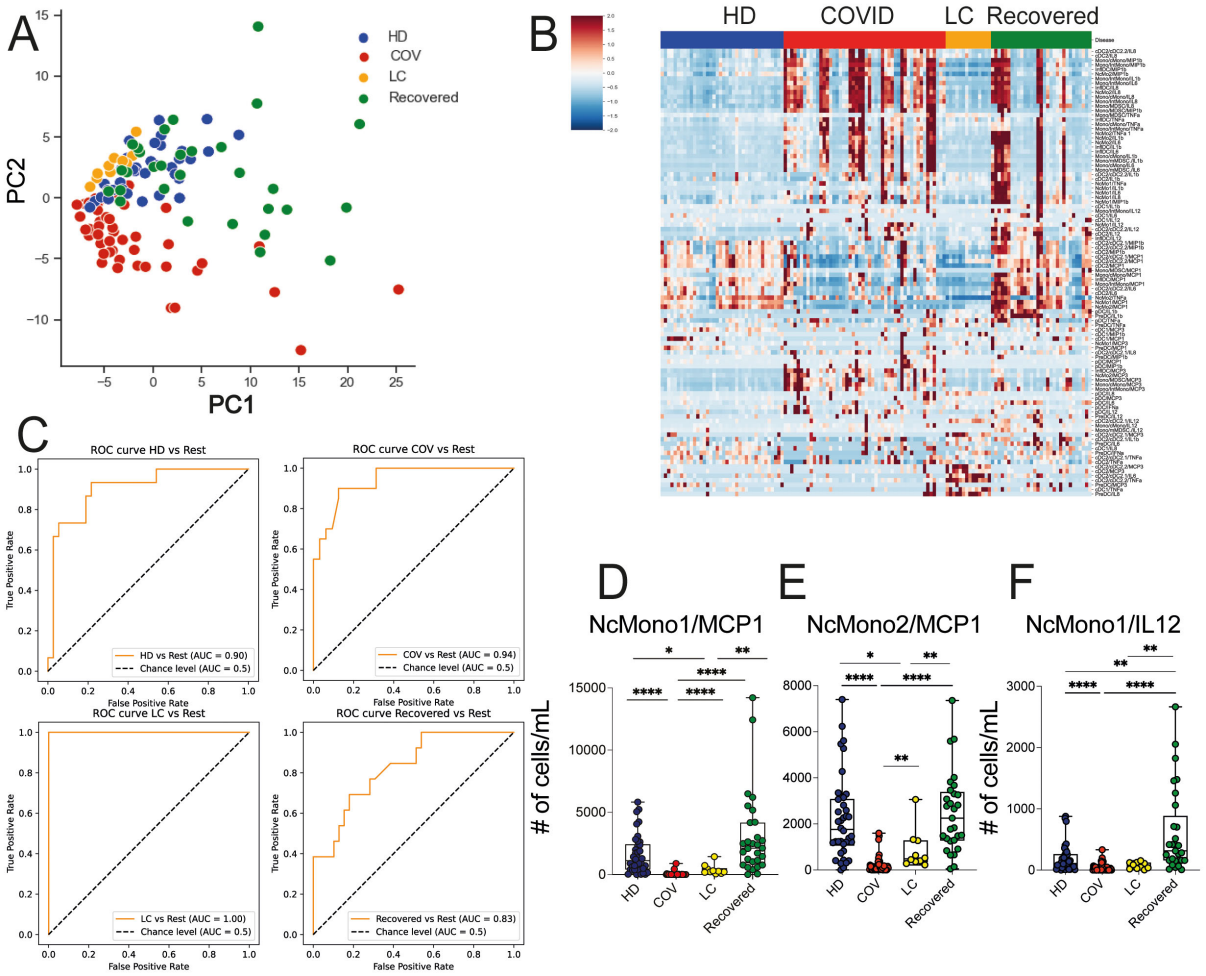
*Ex vivo* production of cytokines and chemokines in myeloid cell populations in HD, LC, COVID and Recovered groups. **(A)** Principal Component Analysis (PCA) and **(B)** heatmap illustrating the standardized values of chemokine- and cytokine-producing myeloid absolute cell counts. For PCA each dot represents one variable. **(C)** XGBoost model was fitted on each training set and performances were evaluated on the respective test sets using the auROC metrics. ROC curves of HD vs Rest (including LC, COVID, Recovered) auROC value 0.90, COV vs Rest (including LC, HD, Recovered) (upper) auROC value 0.94, LC vs Rest (including HD, COVID, Recovered) auROC value 1 and Recovered vs Rest (including LC, COVID, HD) (bottom) auROC value 0.83. **(D–F)** Graph showing the counts obtained by FC data of NcMono1/MCP1, NcMono2/MCP1 and NcMono1/IL12 in HD, COVID, LC and Recovered groups. Box and whiskers represent median of values with interquartile range. COVID (N=50), LC (N=10), HD (N=38), Recovered (N=31). Wilcoxon Rank Sum test for independent groups with the Holm *p-*value correction is shown. * *p<0.05*, ** *p<0.01*, **** *p*<0.0001. No symbol, not significant.

We then proceeded to evaluate the baseline functionality of inflammatory mediator production by myeloid cell populations, including TNF-α, IL-12, IL-6, IL-1β, MCP1, MIP1β and MCP3 through *ex vivo* intracellular staining of whole blood from HD, COVID, LC, and Recovered subjects. We also quantified the absolute cell counts of cells producing the mentioned chemokines and cytokines. To understand whether these immune features were specific for any of the cohorts, we performed global PCA analysis across the four patient groups, which revealed a distinct segregation of the COVID cohort in comparison to LC, HD, and Recovered patients, with the latter three groups displaying a similar distribution (**Figure 3A**). In line, the heatmap visualization (**Figure 3B**), displaying standardized values of chemokine- and cytokine-producing myeloid cell counts, indicates a baseline overproduction of proinflammatory mediators in COVID patients. This overproduction is comparatively less pronounced in the LC group. We further employed an XGBoost classification model, utilizing the absolute cell counts of cytokine- and chemokine-producing myeloid cells as features and disease status as the target variable. The model’s performance, assessed by One-vs-Rest auROC, generated values of 0.94 and 1 for COVID and LC cohorts, respectively, with values of 0.90 for HD and 0.83 for Recovered individuals (**Figure 3C** and **Supplementary Figure 2A**). Variable importance analysis pinpointed the number of NcMono1- and NcMono2- MCP1-producing cells and of NcMono-IL12 producing cells as the top variables distinguishing the four groups (**Supplementary Figure 2A** and **Figures 3D–F**). Notably, absolute numbers of NcMono1 spontaneously producing MCP1 and IL12 were significantly lower in COVID patients compared to the other groups (**Figures 3D, F**), while NcMono2 producing MCP-1 were significantly decreased in COVID compared to HD and Recovered subjects (**Figure 3E**). Individual graphs illustrating the absolute cell counts of the top 10 variables (**Supplementary Figures 2B–H**) demonstrated decreased numbers of inflammatory cytokine- and chemokine- producing cells in COVID patients only for those populations strongly abated during the disease, in line with previous reports, and enhanced production of MCP3 by inflDC (8). These findings suggested a higher basal functional state of myeloid cell populations in COVID patients compared to the LC group, which was more similar to HD or Recovered subjects.

### 3.3 The lower response to immune stimulation observed in COVID-19 patients is detected in also LC patients

**Figure 4:**
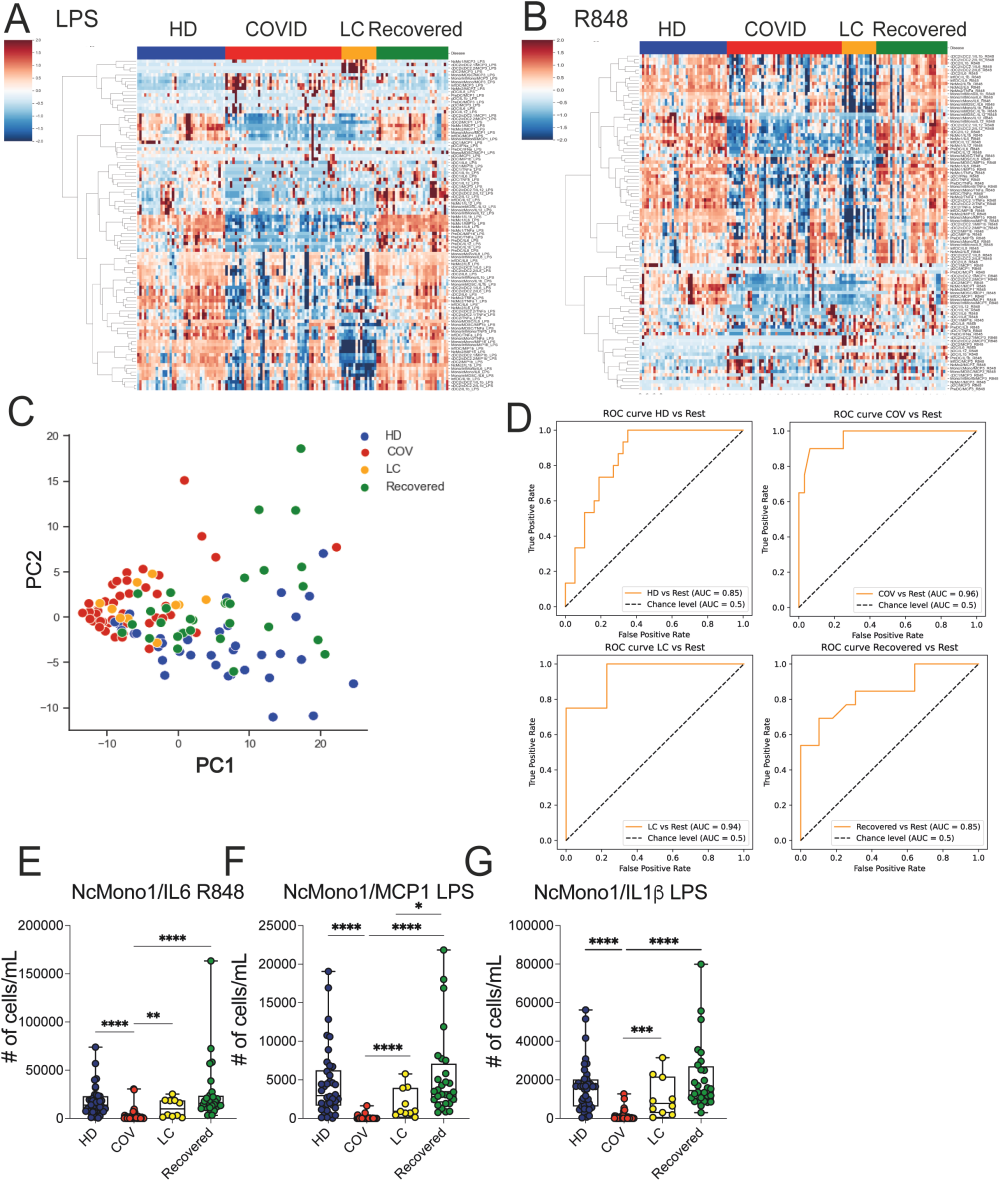
*Ex vivo* analysis of cytokine/chemokine production on myeloid cell populations upon LPS and R848 stimuli. **(A, B)** Heatmap illustrating the standardized values of chemokine- and cytokine-producing myeloid cell counts obtained by FC data upon LPS **(A)** and R848 **(B)** stimulations. **(C)** Principal Component Analysis (PCA) illustrating the standardized values of chemokine- and cytokine-producing myeloid cell counts obtained by FC data upon LPS and R848 stimulation. Each dot represents one variable. **(D)** XGBoost model was fitted on each training set and evaluated their performances on the respective test sets using the auROC metrics. ROC curves of HD vs Rest (including LC, COVID, Recovered) auROC value 0.85, COV vs Rest (including LC, HD, Recovered) (upper) auROC value 0.96, LC vs Rest (including HD, COVID, Recovered) auROC value 0.94 and Recovered vs Rest (including LC, COVID, HD) (bottom) auROC value 0.85. **(E–G)** Graph showing the absolute cell counts of NcMono1/IL6 (R848), NcMono1/MCP1 (LPS), NcMono1/IL1β (LPS) in HD, COVID, LC and Recovered groups. Box and whiskers represent median of values with interquartile range. COVID (N=50), LC (N=10), HD (N=38), Recovered (N=31). Wilcoxon Rank Sum test for independent groups with the Holm *p-*value correction is shown. * *p<0.05*, **p<0.01, *** *p*<0.001 **** *p*<0.0001. No symbol, not significant.

We assessed myeloid cells’ production of inflammatory mediators by mimicking bacterial (LPS) or viral (R848) activation on whole blood using flow cytometry. Freshly obtained whole blood was stimulated for 3h with LPS or R848 in the presence of brefeldin, and cells were then stained with surface markers to define the different cell subsets and then intracellularly to measure cytokine and chemokine production. We then asked whether the immunological features we had measured enabled the separation of the different study groups, with the aim of identifying combination of markers selectively expressed on cells from the LC group. A heatmap displaying standardized values of chemokine- and cytokine-producing myeloid cell counts (**Figures 4A, B**) showed a dampened cytokine- and chemokine-production in both COVID and LC patients, while HD and Recovered subjects displayed a similar pattern in their ability to produce inflammatory mediators upon stimulation. Corroborating this, PCA analysis revealed a partial overlap between the COVID and LC groups, with a clear distinction between LC and HD or Recovered individuals, as displayed in **Figure 4C**. We then employed the same model type as above, utilizing the absolute cell counts of cytokine- and chemokine-producing myeloid cells post-stimulation as features and disease status as the target variable. The model’s performance in classifying disease status, measured by One-vs-rest auROC, yielded values of 0.96 and 0.94 for the COVID and LC cohorts, respectively, and 0.85 for both HD and Recovered individuals (**Figure 4D** and **Supplementary Figure 3A**). Variable importance analysis identified non classical monocytes producing IL-6, MCP1, and IL1β as the top variables distinguishing the four groups, both following TLR2/4 (LPS) or TLR7/8 (R848) stimulation. These variables, assessed individually (**Figures 4E–G**), exhibited significantly lower counts in COVID patients and a trend towards lower counts in LC patients compared to HD or Recovered subjects, indicating defects in the production of these inflammatory cytokines/chemokines during both acute COVID-19 infection and LC syndrome. Additionally, various monocyte cell populations (NcMono, cMono, and IntMono) demonstrated a reduced ability to produce inflammatory cytokines such as IL-1β, IL6, IL12, TNFα (**Supplementary Figures 3C–E, G, H**) and macrophage inflammatory chemokines (MIP1β, MCP1) (**Supplementary Figures 3B–F**) in both COVID and LC patients compared to unexposed HD or Recovered subjects. Overall, inflammatory cytokines and chemokine production by monocytes were lower in both COVID and LC patients, particularly upon stimulation with the viral mimic, suggesting a profound innate immune cell dysregulation occurring during COVID-19, which persists in LC patients.

### 3.4 Depletion of selected T cell subsets and of memory switched CD27+ B cells observed in COVID-19 patients persists in LC patients

**Figure 5:**
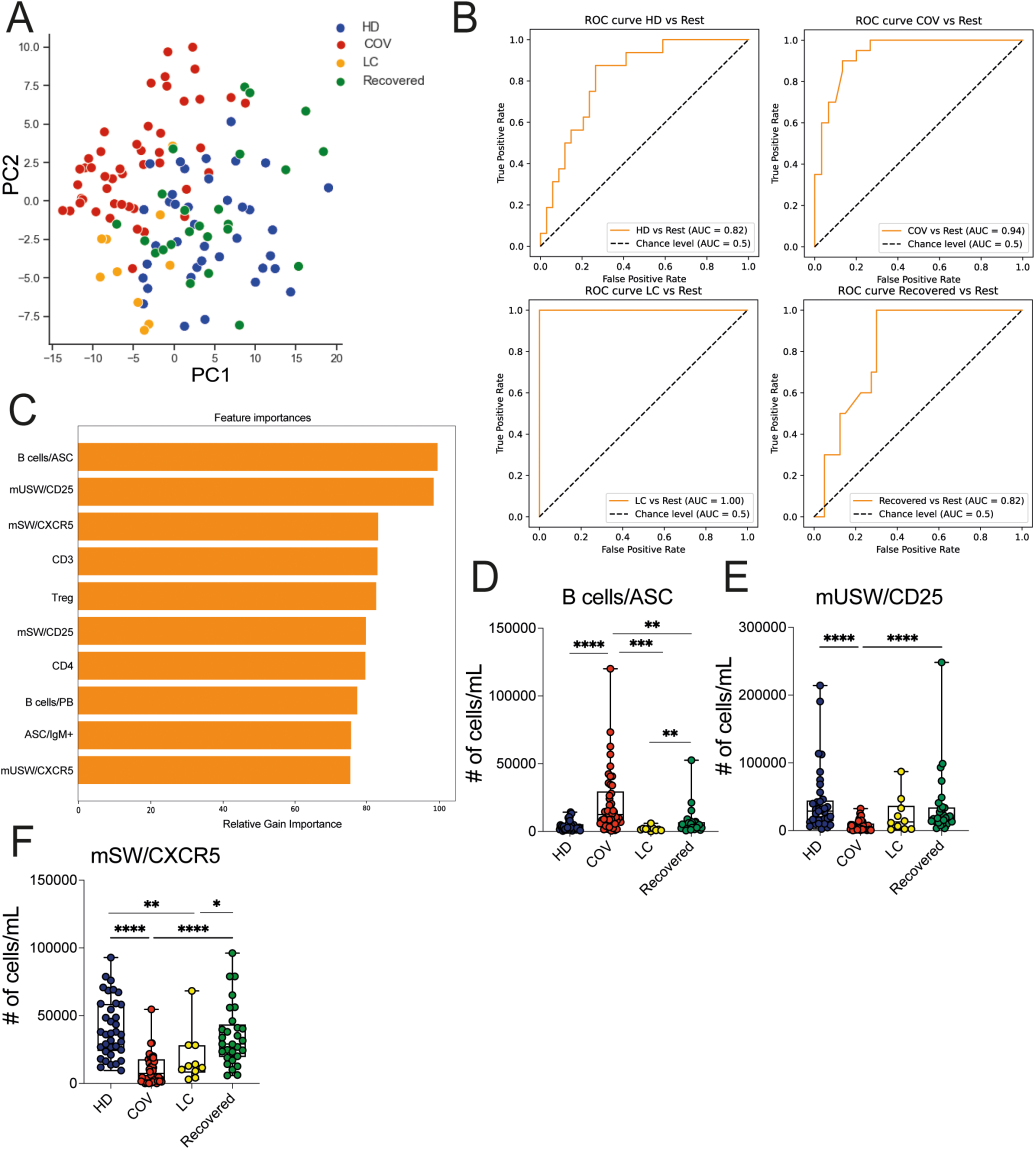
Lymphoid cell population analysis in HD, LC, COVID and Recovered groups. **(A)** Principal Component Analysis (PCA) illustrating the standardized values of lymphocyte immune cell population counts obtained by FC data. Each dot represents one variable. **(B)** XGBoost model was fitted on each training set and evaluated their performances on the respective test sets using the auROC metrics. ROC curves of HD vs Rest (including LC, COVID, Recovered) auROC value 0.82, COV vs Rest (including LC, HD, Recovered) (upper) auROC value 0.94, LC vs Rest (including HD, COVID, Recovered) auROC value 1 and Recovered vs Rest (including LC, COVID, HD) (bottom) auROC value 0.82. **(C)** Graph showing the top (N=10) variables importance of lymphocyte immune cell population counts obtained by FC data as relative gain importance. **(D–F)** Graph showing the counts of Bcells/ASC, mUSW/CD25, mSW/CXCR5 in HD, COVID, LC and Recovered groups. Box and whiskers represent median of values with interquartile range. COVID (N=50), LC (N=10), HD (N=38), Recovered (N=31). Wilcoxon Rank Sum test for independent groups with the Holm *p-*value correction is shown. * p*<0.05*, **p*<0.01*, *** *p*<0.001 **** *p*<0.0001. No symbol, not significant.

We extended the immunological analysis to lymphocyte immune cell populations, including T cells, NK cells, and B cell subsets, employing the gating strategy outlined in **Supplementary Figures 4A–D**, within the HD, COVID, LC, and Recovered groups. Overall, a marked depletion of CD3+ T cells with reduced levels of circulating CD4+, CD8+, and Treg cells was observed in COVID patients compared to HD and recovered individuals (**Supplementary Figure 5A**), consistent with prior findings (14). While total CD3+ T cells, CD4+ T, and Treg cells showed partial recovery, the numbers of circulating CD8+ T cells remained low in LC patients, in line with previous studies (32). Similarly, NK cell counts were lower in COVID patients compared to HD and Recovered subjects (**Supplementary Figure 5B**). MAIT depletion is observed in COVID compared with other groups (**Supplementary Figure 5C**). On the contrary, overall B cell counts remained relatively stable among HD, COVID, LC, and Recovered groups when evaluated as total B, naïve or transitional B cells (**Supplementary Figure 5D**). Memory CD27^+^ B cell counts were lower in COVID patients compared to Recovered and HD subjects and persisted significantly lower in LC respect to HD. Moreover, higher counts of plasmablasts (PB) and plasma cells (PC) were observed in COVID patients compared to LC, HD, and Recovered subjects (**Supplementary Figure 5C**). PCA analysis of the overall counts of lymphocyte immune cell populations, including expression of chemokine receptors and of markers of activation (e.g., CD25, HLA-DR, CXCR5, CCR6, Ki67), showed that the COVID group segregated distinctly, whereas LC patients were separately distributed and in close proximity of HD and Recovered individuals, with the latter two groups being comparably distributed (**Figure 5A**). Consistent with the previous analyses, we trained an XGBoost model using the absolute cell counts of lymphocyte populations, including chemokine receptors and markers of activation as features and disease status as the target variable. The model’s performance, assessed by One-vs-Rest auROC, yielded values of 0.94 and 1 for the COVID and LC cohorts, respectively, and 0.82 for both HD and Recovered individuals (**Figures 5B, C**). Variable importance analysis identified antibody secreting cells (B cells/ASC), CD25-expressing memory unswitched (mUSW), and memory switched CXCR5^+^ B cells as top variables distinguishing the four groups (**Figures 5C–F**). Interestingly, IgM^+^-producing ASC B cells were significantly increased in COVID compared to HD, Recovered, and LC patients, consistent with previous reports (33) (**Supplementary Figure 5E**). In contrast, the frequency of memory switched CXCR5^+^ B cells was significantly reduced in COVID-19 and in LC patients. These data collectively demonstrate a general depletion of T lymphocytes and memory B cells (mainly memory switched B cells) in COVID-19 patients persisting altered in LC patients.

### 3.5 LC patients were distinguished from unexposed HD and recovered subjects by adaptive immune features

**Figure 6:**
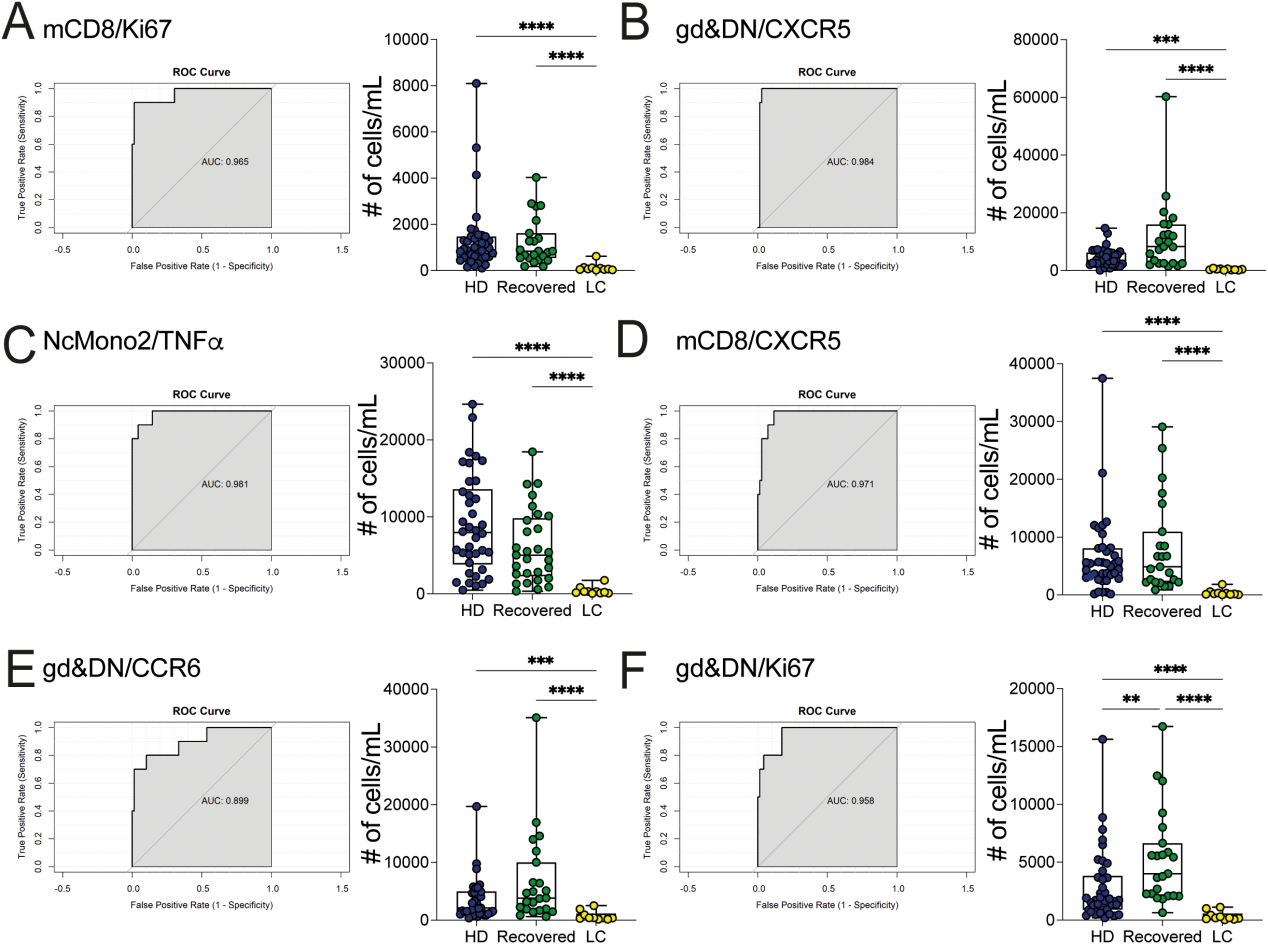
Classification between LC patients and HD/Recovered subjects by local optimum. A GLM model was fitted to find the local optimum and classify LC patients *versus* HD/Recovered subjects. ROC curves (left) and counts obtained by FC data (right) of **(A)** mCD8/Ki67 **(B)** gd&DN/CXCR5, **(C)** NcMono2/TNFα, **(D)** mCD8/CXCR5, **(E)** gd&DN/CCR6, **(F)** gd&DN/Ki67 shown. Graphs (left) show ROC curves and AUC values referring to the top variables. Graphs (right) show all values as count obtained by FC data in HD and Recovered (as group of comparison) and LC group. Box and whiskers represent median of values with interquartile range. Wilcoxon Rank Sum test for independent groups with the Holm *p-*value correction is shown. **p<0.01, *** *p*<0.001 **** *p*<0.0001. No symbol, not significant.

The diagnosis of LC is based on the persistence of symptoms extending beyond 3 months from the initial SARS-CoV2 infection and persisting also until 1- or 2-years, but no definitive markers exist to clearly and objectively identify this condition. Thus, we sought to investigate deeper into the distinctive features of LC patients and to identify potential variables that differentiate LC from Recovered individuals and unexposed HD. Thus, we used all the previously mentioned variables (absolute cell counts of myeloid cell populations, cytokine-chemokine produced by myeloid cells, and T and B lymphocyte subpopulations, including the expression of chemokine receptors and activation markers, and excluding the results from the stimulated cultures). We employed a Binomial Generalized Linear Model (BGLM) for each variable to determine the local optimum, representing the most effective predictor in distinguishing LC from the combined HD/Recovered groups. We included the HD group in this set of analysis due to the limited number of Recovered subjects and the similar distribution of immunological variables between the HD and Recovered subjects. The analysis was based on the area under the receiver operating characteristic curve (auROC) and the accuracy of the Binomial GLM model. We identified 45 markers with auROC values greater than 0.80 (**Figure 6**). The immunological features with the highest GLM accuracy (>0.94) were predominantly represented by adaptive immune elements. Notably, the Ki67-proliferating memory CD8 (mCD8) T cells emerged as the first variable, with lower counts of Ki67^+^- mCD8 T cells observed in LC compared to HD/Recovered individuals (**Figure 6A**). Subsequently, both mCD8- and gamma delta (γδ) & double negative (DN)-CXCR5 and CCR6 expressing cells were found lower in LC than in HD/Recovered individuals (**Figures 6B, D, E**). The same trend of Ki67 expression in mCD8 T cells was observed in gamma delta (γδ) & double negative (DN) cells (**Figure 6F**). Of particular interest, only the number of NcMono2 producing TNF-α was identified as a predictor within the variables derived from the myeloid compartment (**Figure 6C**). These data suggested that LC patients display lower T cell proliferation and reduced expression of T cell activation markers compared to HD and Recovered subjects, as previously shown in **Supplementary Figure 5**, and may serve as specific signature to distinguish LC condition. Despite the model being built on a relatively small cohort of LC patients (N=10), it exhibited the ability to classify LC patients from HD and Recovered subjects, primarily based on adaptive immunological parameters. These findings underscore the significance of monitoring adaptive features in LC subjects, especially focusing on CD8 T cells and gamma delta T cell parameters, which may contribute to distinguishing LC patients from HD/Recovered subjects. Further studies with a larger cohort are crucial to validate these observations.

## 4 Discussion

During infections the innate immune response instinctively becomes activated, and delivers alert signals to all immune components thereby recruiting other innate immune cells and also activating adaptive immune cells. Infection with SARS-CoV-2 is characterized by evasion of the most initial antiviral defense mechanisms mediated by interferon responses (IFNs) (34, 35). Following this defense leak, inflammation can take a serious turn, leading to cytokine storm and to extended tissue damage, or to prolonged recurrent relapses as in the case of LC. LC syndrome is defined by persisting symptoms that cannot be explained by an alternative diagnosis and affect both adults and children (17). While some advances have been made in defining the immunological factors associated with LC syndrome, we are still far from understanding the mechanisms linking SARS-CoV-2 infection to the symptoms described in LC. The most commonly observed symptoms include persistent physical (i.e., fatigue, dyspnea, chest pain/tightness, cough) and mental symptoms (i.e., sleep disturbances, depression, anxiety, cognitive impairment). Indeed, patients with LC showed clinical abnormalities such as dyspnea at rest, exertion, despite normal physical or cardiopulmonary function tests and negative PCR tests (36). Nowadays, LC is diagnosed mainly based on those clinical symptoms, rather than on a set of immunological markers. Here, we searched for immunological signatures which could uniquely identify LC patients, performing a study on the immunological profile of individuals who had been infected by SARS-CoV-2 and subjects who had recovered from the infection, with particular focus on the differences between HD/Recovered subjects and LC patients. We measured innate and adaptive immune parameters, such as absolute myeloid, T and B cell counts, cytokine and chemokine production by myeloid cells, and the expression of chemokine receptors and activation markers in lymphocytes, in fresh blood samples from a cohort of patients with LC, (hospitalized) acutely infected, recovered, or healthy uninfected donors, in search for a set of immunological markers which most strongly associated with LC, both to identify novel diagnostic tools and to better understand the pathophysiology underlying symptom persistence and recurrence. In line with previous studies, we found that COVID-19 is characterized by dysregulation of both myeloid and lymphoid immune cells, with a higher level of inflammation deriving from innate immune cells, and alterations in the distribution of immune cell subsets (37–39). Interestingly, our data suggested that LC patients are characterized by a different composition of innate and adaptive immune cells compared to COVID-19 patients, showing more similarities with HD and Recovered subjects. All myeloid cell subsets are strongly reduced in the peripheral blood of COVID-19 patients, particularly non classical monocytes which nearly disappear during acute infection. In contrast, we observed that most of the myeloid immune populations, including both classical and non-classical monocytes in LC patients, recovered levels similar to those in HD and Recovered subjects. Classical monocytes represent a transient population of cells newly released by the bone marrow, which undergo one of two destinies: *i)* migration in tissues, to replenish the resident monocyte-derived cell population, or *ii)* differentiation into non-classical monocytes (40). Non-classical monocytes patrol the endothelial lining and are considered to be endothelial “caretakers”, promoting resolution of inflammation (41), although they are also involved in inducing T cell activation and proliferation and in fostering adhesion of neutrophils to endothelial cells. Our data showed that while COVID-19 patients exhibited an overproduction of cytokines and chemokines *ex vivo* by non-classical monocytes, as previously reported (42), this was not observed in LC patients. However, when exposed to bacterial or viral mimics, non-classical monocytes from LC subjects displayed a significantly reduced ability to produce inflammatory cytokines and chemokines, similar to what was observed in COVID-19 patients. One potential driver of non-classical monocyte dysfunction in LC patients may be a persistent SARS-CoV-2 reservoir in host organs (24, 43), which continuously stimulates tissue-myeloid cells, rendering them unable to respond to further stimuli.

Adaptive immune responses are also deeply dysregulated in acute SARS-CoV2 infection. This perturbation affected both T and B cell populations (44). Consistent with our data, the acute phase of SARS-CoV-2 infection is characterized by T cell depletion, while an increase of CD4^+^ T cells is observed in LC (45). Some authors showed that CD4^+^ T cells from LC patients preferentially expressed homing receptors associated with migration to inflamed tissues (45), suggesting a direct impact of virus persistence in organs in balancing the phenotype and the migration of T cells. The remodeling of T cell dynamics, showing an increase in the T cell exhaustion phenotype, appeared predominantly in LC and dependent on infection severity (46). As confirmed by others, we have shown an increase of Treg cells in number (45) in LC patients. We proposed that the state of chronic inflammation, characterizing LC patients, may promote the expansion of regulatory T cells, as observed in other chronic infections (47, 48), as a response to limit the excessive immune activation and tissue damage. Extrafollicular B cell activation is also a dominant feature of severe and critical COVID-19 infection, often associated with new autoreactive Abs, as previously reported in studies in autoimmunity (49–53). In line with our observations, a profound dysregulation of B cells, with the expansion of ASC B cells (53) and PB cells is observed in SARS-CoV-2 infection (54, 55). The vast majority of people recovering from infection developed T and B cell memory responses. The memory B cells, detected in recovered patients up to 6 months post-infection, produced PC secreting SARS-CoV-2 neutralizing Abs, suggesting that memory B cells can contribute to the production of protective Abs when restimulated (56). Our data suggested a reduction of PB and PC in the LC group compared to COVID-19 patients. It is likely that during the acute phase of infection, the significant antibody production required to eradicate the virus induces a profound reshaping of the PB, PC, and ASC B cell compartments. The persistence of the SARS-CoV-2 virus in LC patients may mimic a state of chronic infection, thus restoring the general balance of PB, PC, and ASC B cells. Although we find a reduction of PB and PC cell numbers, a recent study demonstrated exaggerated humoral responses with higher antibody responses directed against non-SARS-CoV-2 viral pathogens, particularly Epstein–Barr virus among participants with LC (23). Collectively, multiple factors contribute to LC, including chronic infection or viral reactivation (57–60), unresolved tissue damage (61), autoimmunity (62–64), complement dysregulation (65), immune exhaustion (46) and inflammation (18, 66, 67). The finding that the most robust predictor for LC involves a diverse combination of biomarkers underscores the wide array of host response pathways that become activated during this syndrome, and which may drive symptom chronicity. The immunological signatures which define the different disease states may be used to guide diagnostic procedures from samples of whole blood, and to personalize treatment regimens. To date, no clinical parameters can identify LC condition and the laboratory-based diagnosis is still lacking.

Although our study was limited by the low number of LC patients, we present evidence of an immunological signature, specifically observed in patients with LC, compared to HD/Recovered subjects, characterized by the lower proliferative capacity of memory CD8 T cells, evaluated by Ki67 expression and lower activation markers on both memory CD8 T cells and γδ T cells, including the expression of homing and resident receptors such as CXCR5 and CCR6, respectively. Notably, CXCR5^+^ CD8 T cells have been associated with various chronic viral infections (68), with a crucial role in controlling the viral replication and eliminating the viral reservoir. In the animal model, during infection with Influenza A virus CXCR5+CD8+ T cells arise, actively influencing humoral immunity by shaping antibody responses even in non-follicular microenvironments (69). These cells have also been involved in the control of chronic human HBV infection, showing a clear correlation with favorable clinical outcomes (70). In line with these data, some authors demonstrated that a proportion of CD8^+^ T cells expressing CXCR5 increased in SARS-CoV-2 infected individuals (71) and enriched in surviving COVID-19 patients compared to those who succumbed to the infection, thereby conferring protection against SARS-CoV-2 infection (72). It is likely that the lower accumulation of CXCR5^+^ CD8 T cells observed in the LC patient cohort may fail to control the viral replication, thus allowing the chronic persistence of SARS-CoV2. We propose, therefore, that among the identified immunological markers, tracking the expression of CXCR5^+^ on CD8 and on γδ T cells in the periphery may help in identifying LC individuals. Overall, our results identified an immunological signature associated with the LC phenotype that may be useful for characterizing the pathogenesis and for defining a diagnostic tool for LC syndrome.

## Data Availability

The raw data supporting the conclusions of this article will be made available by the authors, without undue reservation.

## Supplementary material

Supplementary Figure 1
**(A)** Representative gating strategy of one COVID patient on myeloid cell populations. **(B)** Graph showing the top (N=10) variables importance of myeloid cell population counts as relative gain importance. **(C-I)** Graphs showing the counts of **(C)** IntMono **(D)** cMono, **(E)** cDC2.2, **(F)** NcMono2, **(G)** cDC2, **(H)** InflDC and **(I)** MDSC in HD, COVID, LC and Recovered groups. Box and whiskers represent median of values with interquartile range. COVID (N=50), LC (N=10), HD (N=38), Recovered (N=31). Wilcoxon Rank Sum test for independent groups with the Holm p-value correction is shown. * p<0.05, **p<0.01, ***p<0.001 **** p<0.0001. No symbol, not significant.

Supplementary Figure 2
**(A)** Graph showing the top (N=10) variables importance of ex vivo cytokine/chemokine production on myeloid cell populations as counts and as relative gain importance. B-H) Graphs showing the counts of **(B)** Infl/IL12 **(C)** preDC/IL1·, **(D)** InflDC/MCP3, **(E)** cDC2/IL6, **(F)** pDC/IL1·, **(G)** cDC1/IL6, (H) NcMono2/TNF· in HD, COVID, LC and Recovered groups. Box and whiskers represent median of values with interquartile range. COVID (N=50), LC (N=10), HD (N=38), Recovered (N=31). Wilcoxon Rank Sum test for independent groups with the Holm p-value correction is shown. * p<0.05, **p<0.01, *** p<0.001 **** p<0.0001. No symbol, not significant..

Supplementary Figure 3
**(A)** Graph showing the top (N=10) variables importance of ex vivo cytokine/chemokine production upon LPS and R848 stimulation on myeloid cell populations as counts. The graph shows the relative gain importance. **(B-H)** Graphs showing the counts of **(B)** NcMono1/MCP1 R848 **(C)** cMono/IL1· LPS, **(D)** NcMono1/IL1··R848, **(E)** cMono/IL12 R848, **(F)** cMono/MIP1· R848 **(G)** cMono/TNF··LPS, **(H)** IntMono/IL6 LPS in HD, COVID, LC and Recovered groups. Box and whiskers represent median of values with interquartile range. COVID (N=50), LC (N=10), HD (N=38), Recovered (N=31). Wilcoxon Rank Sum test for independent groups with the Holm p-value correction is shown. * p<0.05, **p<0.01, *** p<0.001 ****p<0.0001. No symbol, not significant..

Supplementary Figure 4
**(A)** Representative common progressive gating strategy of both myeloid and lymphoid cell populations of one COVID patient. **(B, C)** Representative gating strategy and T and NK cell populations and activation markers of COVID patient. **(D)** Representative gating strategy of B cell populations and activation markers of COVID patient..

Supplementary Figure 5
**(A)** Graphs showing the counts obtained by FC data of CD3 T, CD8 T, CD4 T cells and Treg (CD127lowCD25+), **(B)** NK (CD56+) cells **(C)** MAIT cells, **(D)** B cells, naïve (n)B cells, Transitional and memory CD27+ B cells, plasmablasts (PB) and plasma cells (PC). **(E)** Graphs showing the counts obtained by FC data of two top variables (referring to **Figure 5**) of mSW/CD25 and ASC/IgM+ B cells. Box and whiskers represent median of values with interquartile range. COVID (N=50), LC (N=10), HD (N=38), Recovered (N=31). Wilcoxon Rank Sum test for independent groups with the Holm p-value correction is shown. * p<0.05, ** p<0.01, *** p<0.001 **** p<0.0001. No symbol, not significant.
