# Supplementary figures and images for: Identification of an immunological signature of long COVID syndrome
Source: Front Immunol. 2025 Jan 8;15:1502937. doi: 10.3389/fimmu.2024.1502937 (PMC11750999; doi:10.3389/fimmu.2024.1502937)

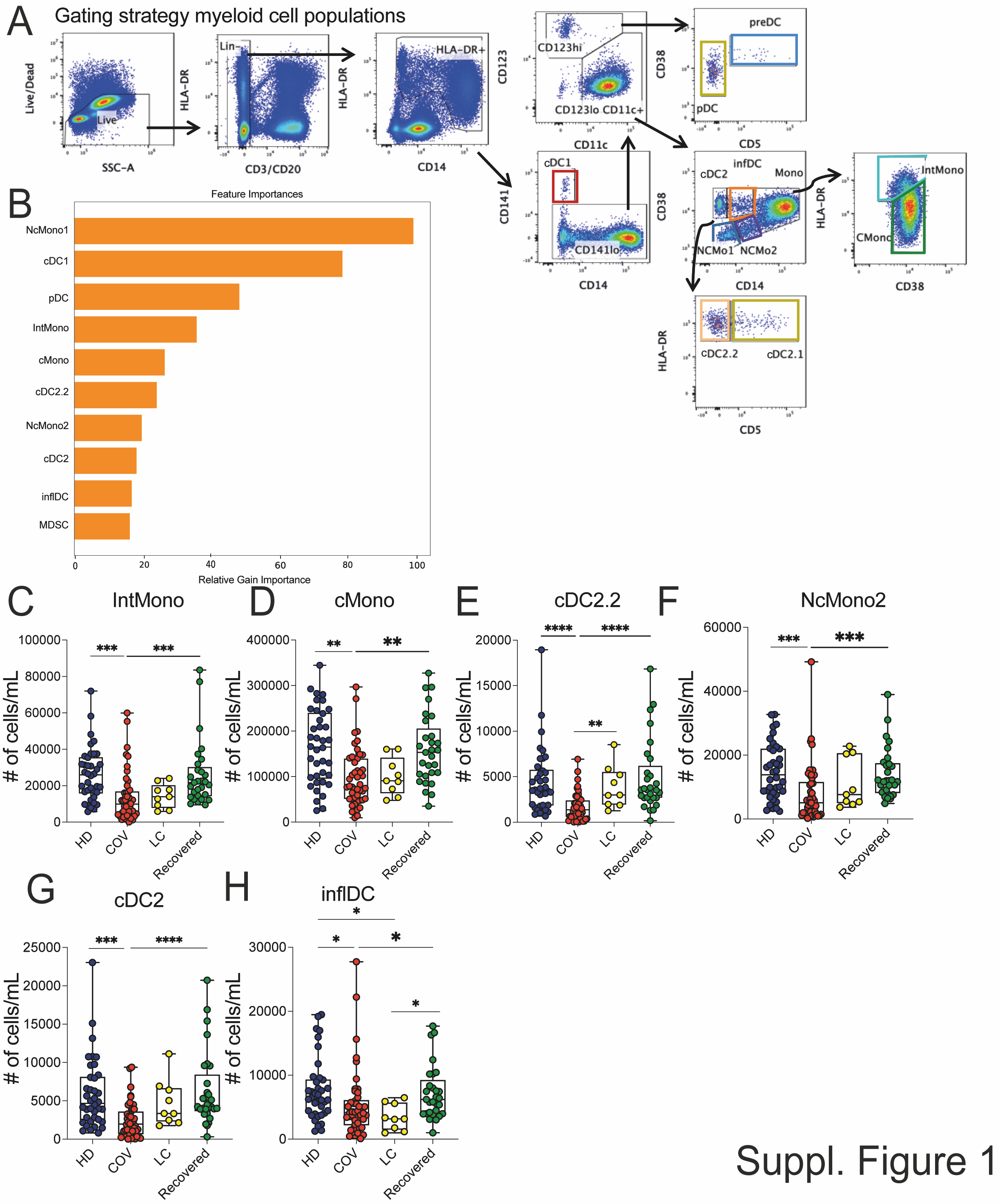

Supplement: Supplementary Figure 1 — (A) Representative gating strategy of one COVID patient on myeloid cell populations. (B) Graph showing the top (N=10) variables importance of myeloid cell population counts as relative gain importance. (C-I) Graphs showing the counts of (C) IntMono (D) cMono, (E) cDC2.2, (F) NcMono2, (G) cDC2, (H) InflDC and (I) MDSC in HD, COVID, LC and Recovered groups. Box and whiskers represent median of values with interquartile range. COVID (N=50), LC (N=10), HD (N=38), Recovered (N=31). Wilcoxon Rank Sum test for independent groups with the Holm p-value correction is shown. * p<0.05, **p<0.01, ***p<0.001 **** p<0.0001. No symbol, not significant. [file Image1.tiff]

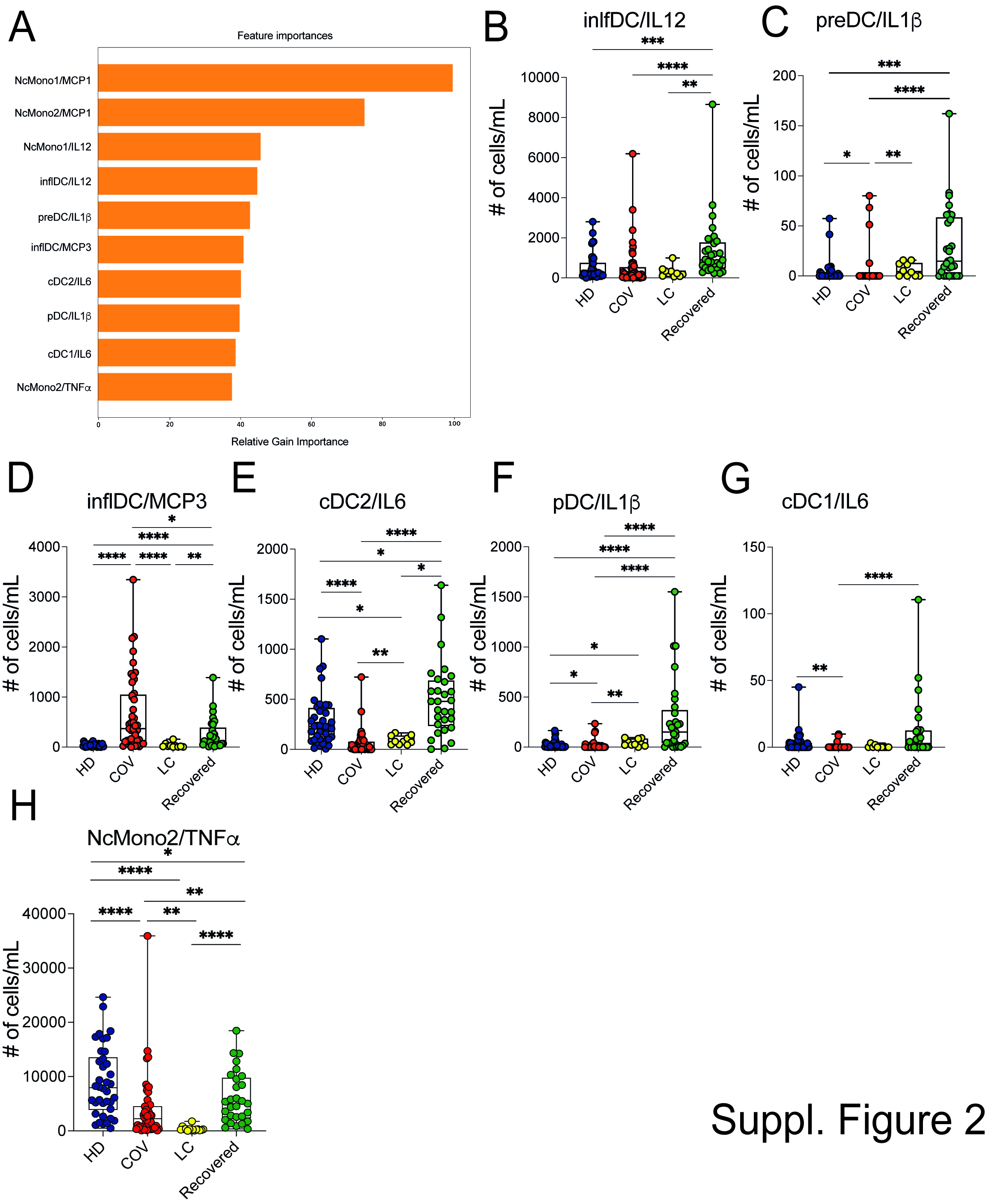

Supplement: Supplementary Figure 2 — (A) Graph showing the top (N=10) variables importance of ex vivo cytokine/chemokine production on myeloid cell populations as counts and as relative gain importance. B-H) Graphs showing the counts of (B) Infl/IL12 (C) preDC/IL1·, (D) InflDC/MCP3, (E) cDC2/IL6, (F) pDC/IL1·, (G) cDC1/IL6, (H) NcMono2/TNF· in HD, COVID, LC and Recovered groups. Box and whiskers represent median of values with interquartile range. COVID (N=50), LC (N=10), HD (N=38), Recovered (N=31). Wilcoxon Rank Sum test for independent groups with the Holm p-value correction is shown. * p<0.05, **p<0.01, *** p<0.001 **** p<0.0001. No symbol, not significant.. [file Image2.tif]

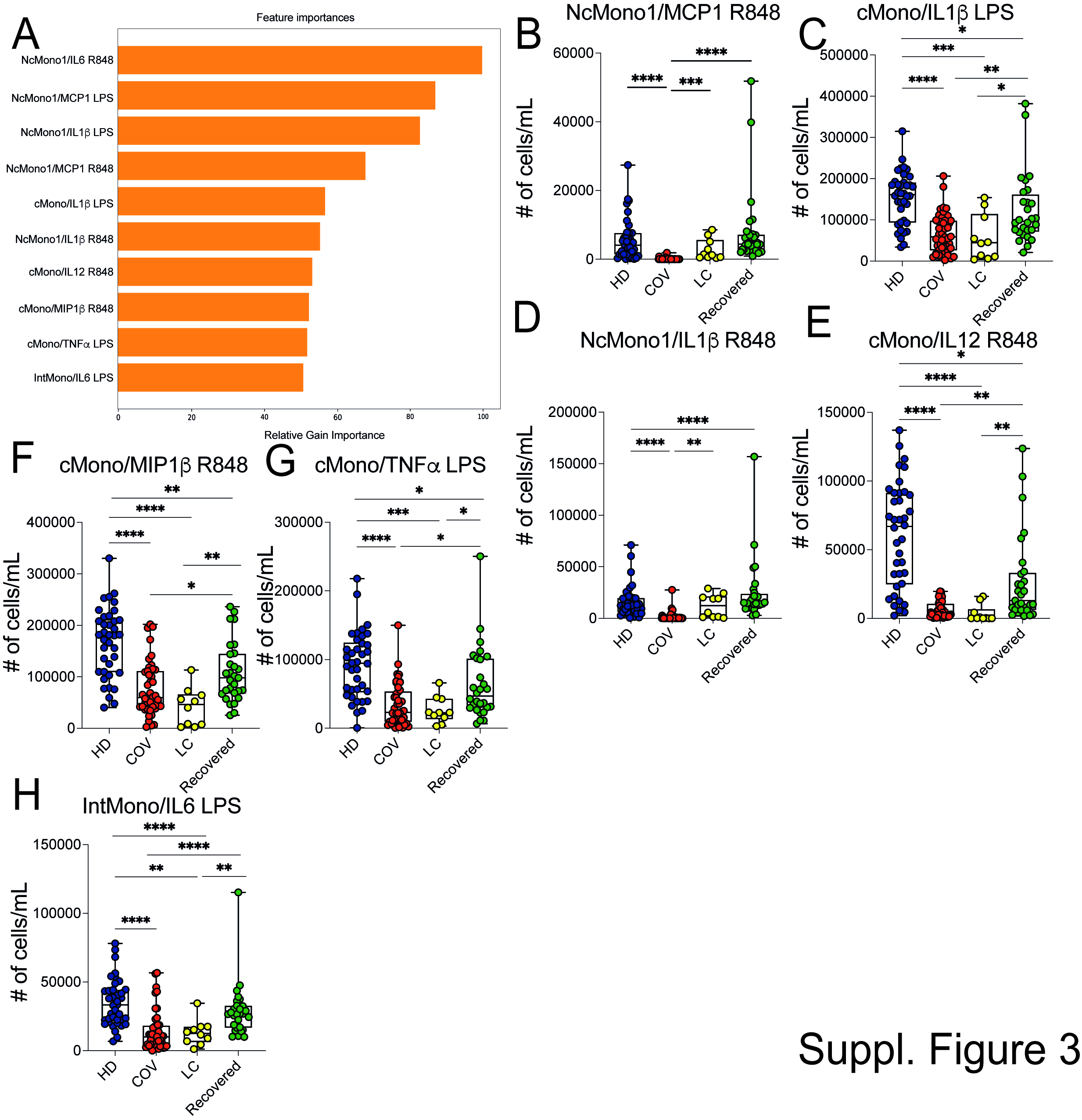

Supplement: Supplementary Figure 3 — (A) Graph showing the top (N=10) variables importance of ex vivo cytokine/chemokine production upon LPS and R848 stimulation on myeloid cell populations as counts. The graph shows the relative gain importance. (B-H) Graphs showing the counts of (B) NcMono1/MCP1 R848 (C) cMono/IL1· LPS, (D) NcMono1/IL1··R848, (E) cMono/IL12 R848, (F) cMono/MIP1· R848 (G) cMono/TNF··LPS, (H) IntMono/IL6 LPS in HD, COVID, LC and Recovered groups. Box and whiskers represent median of values with interquartile range. COVID (N=50), LC (N=10), HD (N=38), Recovered (N=31). Wilcoxon Rank Sum test for independent groups with the Holm p-value correction is shown. * p<0.05, **p<0.01, *** p<0.001 ****p<0.0001. No symbol, not significant.. [file Image3.tif]

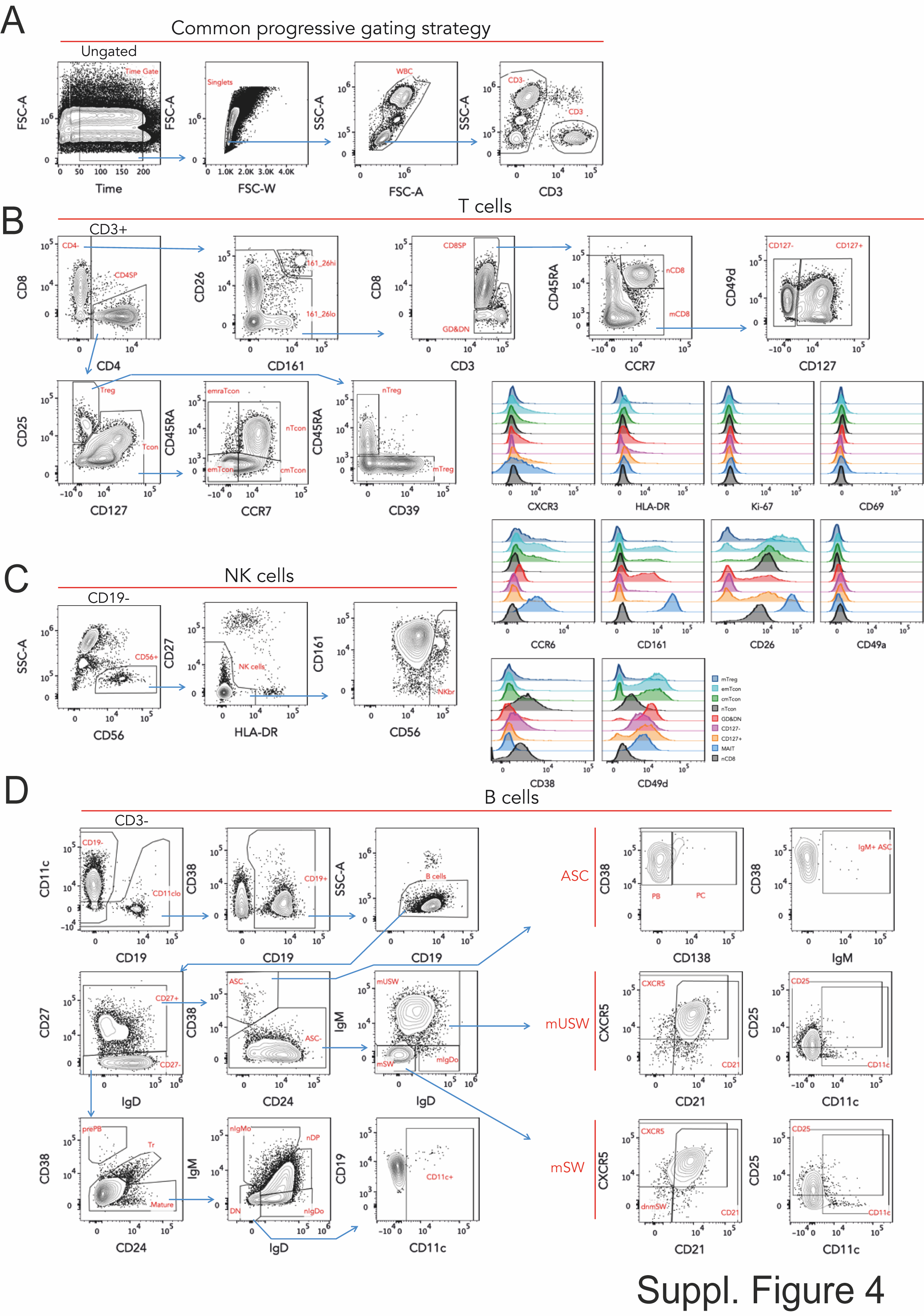

Supplement: Supplementary Figure 4 — (A) Representative common progressive gating strategy of both myeloid and lymphoid cell populations of one COVID patient. (B, C) Representative gating strategy and T and NK cell populations and activation markers of COVID patient. (D) Representative gating strategy of B cell populations and activation markers of COVID patient.. [file Image4.tif]

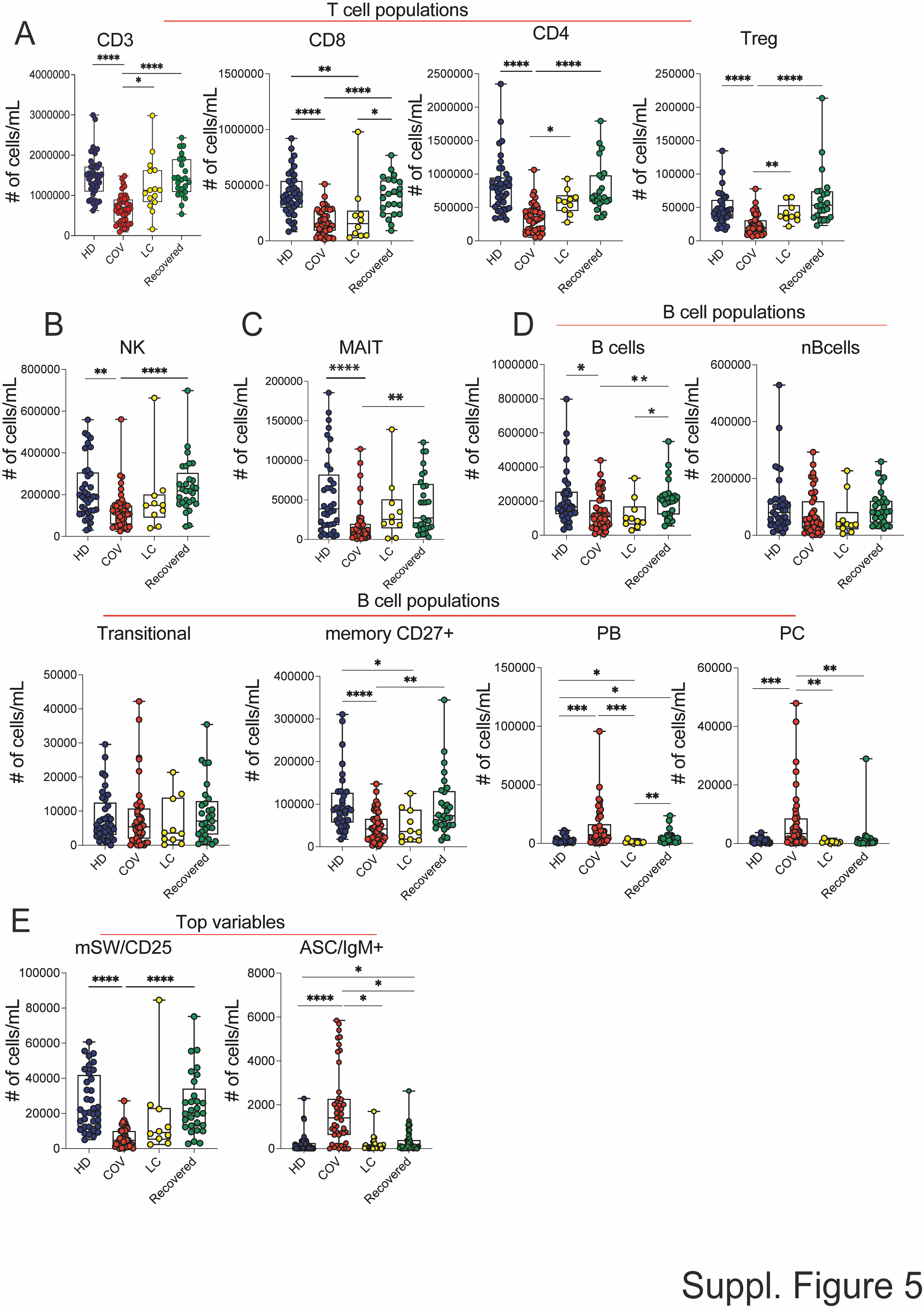

Supplement: Supplementary Figure 5 — (A) Graphs showing the counts obtained by FC data of CD3 T, CD8 T, CD4 T cells and Treg (CD127lowCD25+), (B) NK (CD56+) cells (C) MAIT cells, (D) B cells, naïve (n)B cells, Transitional and memory CD27+ B cells, plasmablasts (PB) and plasma cells (PC). (E) Graphs showing the counts obtained by FC data of two top variables (referring to Figure 5 ) of mSW/CD25 and ASC/IgM+ B cells. Box and whiskers represent median of values with interquartile range. COVID (N=50), LC (N=10), HD (N=38), Recovered (N=31). Wilcoxon Rank Sum test for independent groups with the Holm p-value correction is shown. * p<0.05, ** p<0.01, *** p<0.001 **** p<0.0001. No symbol, not significant. [file Image5.tif]
