# Supplementary material for: Identification of an immunological signature of long COVID syndrome
Source: Front Immunol. 2025 Jan 8;15:1502937. doi: 10.3389/fimmu.2024.1502937 (PMC11750999; doi:10.3389/fimmu.2024.1502937)
Supplement: Supplementary file 6 [file Table1.docx]

Supplementary Material

# Supplementary Material

The list of all antibodies reported specifies: antibody name, fluorochrome, clone, company and the dilution factor used for each experiment.

| **Antibody** | **Fluorochrome** | **Clone** | **Company** | **Titer** |
| --- | --- | --- | --- | --- |
| CD3 | APC-Vio770 | REA613 | Miltenyi | 1:240 |
| CD14 | PE-Cy7 | RMO52 | Coulter | 1:100 |
| CD16 | Pacific Blue | 3G8 | Bect.Dick. | 1:100 |
| CD19 | PE | REA675 | Miltenyi | 1:120 |
| CD45 | FITC | KC56 | Coulter | 1:200 |
| CD56 | PE-CF594 | NCAM16.2 | Bect.Dick. | 1:60 |
| CD123 | BV605 | 9F5 | Bect.Dick. | 1:60 |
| HLA-DR | BV785 | G46-6 | Bect. Dick. | 1:100 |

| **Antibody** | **Fluorochrome** | **Clone** | **Company** | **Titer** |
| --- | --- | --- | --- | --- |
| CD3 | PE-Cy5 | UCHT1 | Coulter | 1:60 |
| CD14 | Fitc | REA599 | Miltenyi | 1:100 |
| IL-1β | PE | AS-10 | Bect.Dick. | 1:120 |
| MCP-3 | PE | REA328 | Miltenyi | 1:100 |
| IL-6 | PE-CF594 | MQ2-13A5 | Bect.Dick. | 1:400 |
| IL-8 | PE-CF594 | G265-8 | Bect.Dick. | 1:100 |
| CD20 | PE-Cy5 | B9E9 (HRC20) | Coulter | 1:30 |
| CD5 | PE-Cy7 | BL1a | Coulter | 1:100 |
| IFN-α | APC | LT27:295 | Miltenyi | 1:60 |
| MCP-1 | APC | REA248 | Miltenyi | 1:30 |
| MIP-1β | APC-Al700 | D21-1351 | Bect.Dick. | 1:100 |
| CD141 | APC-Vio770 | AD5‐14H12 | Miltenyi | 1:120 |
| IL-12p40/70 | BV-421 | C8.6 | Pharmingen | 1:400 |
| GM-CSF | Pacific Blue | BVD2-21C11 | Biolegend | 1:30 |
| CD38 | BV-480 | HIT2 | Bect.Dick. | 1:30 |
| CD123 | BV605 | 9F5 | Bect.Dick. | 1:30 |
| CD56 | BV-650 | NCAM16.2 | Bect.Dick. | 1:120 |
| TNF-α | BUV-395 | MAb11 | Bect.Dick. | 1:100 |
| CD11c | BUV-661 | B-ly6 | Bect.Dick. | 1:200 |
| HLA-DR | BV785 | G46-6 | Bect.Dick. | 1:30 |
| Live Dead | Promo Fluor 840 | NA | Promokine | 1:10.000 |

| **Antibody** | **Fluorochrome** | **Clone** | **Company** | **Titer** |
| --- | --- | --- | --- | --- |
| CD45 | BB700 | HI30 | Bect.Dick. | 1:100 |
| CD40 | PE | 5C3 | Pharmingen | 1:60 |
| CD86 | PE-CF594 | 2331 (FUN-1) | Bect.Dick. | 1:120 |
| CD274 | APC | PDL1.3.1 | Coulter | 1:100 |
| CD80 | APC-Al700 | L307.4 | Bect.Dick. | 1:60 |
| CD16 | Pacific Blue | 3G8 | Bect.Dick. | 1:100 |
| CD83 | BV650 | HB15e | Bect.Dick. | 1:60 |
| Ki-67 | BV785 | B56 | Bect.Dick. | 1:30 |
| HLA-DR | BUV395 | G46-6 | Bect.Dick. | 1:50 |

| **Antibody** | **Fluorochrome** | **Clone** | **Company** | **Titer** |
| --- | --- | --- | --- | --- |
| CD3 | BUV-395 | UCHT1 | Bect.Dick. | 1:30 |
| CD25 | BB515 | 2A3 | Bect.Dick. | 1:80 |
| CD49d | PE-CF594 | 9F10 | Bect.Dick. | 1:60 |
| CD69 | BB700 | FN50 | Bect.Dick. | 1:100 |
| CD49a | PE | SR84 | Pharmingen | 1:30 |
| CD8 | iFluor594 | SK1 | AAT Bioquest | 1:100 |
| CD127 | PE-Cy5 | R34.34 | Coulter | 1:150 |
| CCR7 | PE-Cy7 | G043H7 | Biolegend | 1:80 |
| CXCR3 | APC-Al647 | G025H7 | Biolegend | 1:30 |
| CXCR5 | APC-R700 | RF8B2 | Bect.Dick. | 1:60 |
| HLA-DR | APC-Vio770 | REA805 | Miltenyi | 1:100 |
| CD161 | BV-421 | HP-3G10 | Biolegend | 1:40 |
| CD45RA | BV-480 | HI100 | Bect.Dick. | 1:150 |
| CCR6 | BV605 | 11A9 | Bect.Dick. | 1:20 |
| Ki-67 | BV785 | B56 | Bect.Dick. | 1:30 |
| CD38 | BUV496 | HIT2 | Bect.Dick. | 1:60 |
| CD26 | BUV661 | M-A261 | Bect.Dick. | 1:60 |
| CD4 | iFluor810 | RPA-T4 | AAT Bioquest | 1:200 |
| Live Dead | Promo Fluor 840 | NA | Promokine | 1:10.000 |

| **Antibody** | **Fluorochrome** | **Clone** | **Company** | **Titer** |
| --- | --- | --- | --- | --- |
| CD24 | Fitc | ML5 | Bect.Dick. | 1:30 |
| CD294 (CRTh2) | Fitc | BM16 | Coulter | 1:80 |
| CD25 | PE-CF594 | M-A251 | Pharmingen | 1:60 |
| CD45RA | BB700 | 5H9 | Bect.Dick. | 1:120 |
| CD21 | PE | B-ly4 | Bect.Dick. | 1:100 |
| CD161 | PE | 191B8 | Coulter | 1:60 |
| CD8 | iFluor 594 | SK1 | AAT Bioquest | 1:150 |
| CD127 | PE-Cy5 | R34.34 | Coulter | 1:120 |
| CD34 | PE-Cy5 | 581 | Coulter | 1:60 |
| CD138 | PE-Cy5 | B-B4 | Coulter | 1:30 |
| CD27 | PE-Cy5.5 | 1A4CD27 | Coulter | 1:100 |
| CD14 | PE-Cy5.5 | RMO52 | Coulter | 1:200 |
| IgD | PE-Cy7 | IA6-2 | Pharmingen | 1:60 |
| TCRVα7.2 | PE-Cy7 | 3C10 | Biolegend | 1:100 |
| CD123 | APC | 6H6 | eBioscience | 1:60 |
| TCRγ/δ | APC | 11F2 | Miltenyi | 1:30 |
| CXCR5 | APC-R700 | RF8B2 | Bect.Dick. | 1:60 |
| CD28 | APC-Vio770 | REA612 | Miltenyi | 1:120 |
| CD141 | APC-Vio770 | AD5‐14H12 | Miltenyi | 1:120 |
| IgM | BV421 | G20-127 | Bect.Dick. | 1:120 |
| CD56 | BV421 | NCAM16.2 | Bect.Dick. | 1:50 |
| CD279 | BV480 | EH12.1 | Bect.Dick. | 1:30 |
| CLA | BV605 | HECA-452 | Bect.Dick. | 1:30 |
| CD19 | BV650 | HIB19 | Bect.Dick. | 1:30 |
| HLA-DR | BV785 | G46-6 | Bect.Dick. | 1:30 |
| CD3 | BUV-395 | UCHT1 | Bect.Dick. | 1:30 |
| CD38 | BUV496 | HIT2 | Bect.Dick. | 1:60 |
| CD11c | BUV-661 | B-ly6 | Bect.Dick. | 1:200 |
| CD4 | iFluor810 | RPA-T4 | AAT Bioquest | 1:100 |
| Live Dead | Promo Fluor 840 | NA | Promokine | 1:10.000 |

## Supplementary Tables

**­Supplementary Table 1. Clinical information of COVID-19 patients.**

**­Supplementary Table 2. Clinical information of LC patients.**

**Supplementary Table 2**

Socio-demographic, clinical and study features of LC study participants.

**N** 10

**Place of recruitment**

Hospital 10 (100%)

Non-hospital 0

**Days Post-COVID19 infection** 279 **±** 201

**Sex**

Male 8 (80%)

Female 2 (20%)

**Age (y.o)** 64.5 ± 8.9

**BMI (kg/m^2^)**

Normal weight 3 (30%)

Overweight 1 (10%)

Obese 2 (20%)

Missing 4 (40%)

**Number of COVID-19 high risk comorbidities**

0 2 (20%)

1 5 (50%)

2 3 (30%)

3 or more 0

**Ventilation during COVID-19 hospitalization**

HFNC/CPAP/NIV 2 (20%)

NIV 2 (20%)

CPAP 2 (20%)

VMK 1 (10%)

None 3 (30%)

**Mini-mental state examination (MMSE score)**

Questionably significant (25-30) 3 (30%)

Mild (20-25) 6 (60%)

Moderate (10-20) 1 (10%)

Severe (0-10) 0

**Fatigue Assessment Scale (FAS score)***

Normal (<22) 7 (70%)

Mild (22-34) 3 (30%)

Severe (>34) 0

**Modified MRC scale of dyspnoea (mMRC score)***

Grade 0 3 (30%)

Grade 1 5 (50%)

Grade 2 2 (20%)

Grade 3 0

Grade 4 0

**COVID-19 Vaccination**

Yes 6 (60%)

No 4 (40%)

**COVID-19 Vaccine Type**

Pfizer/BioNTech (BNT162b2) mRNA 5 (83%)

Moderna mRNA 0

AstraZeneca 0

Janssen (Johnson & Johnson) 1 (17%)

Missing 0

**LC:** Long COVID-19; **BMI**: Body Mass Index; BMI categories defined as: <25 Kg/m^2^ normal or underweight, 25-29 Kg/m^2^ overweight, >30 Kg/m^2^ obese; **High risk comorbidities** are defined as listed by the WHO Clinical Management Guidelines and include: cardiovascular disease (including hypertension), chronic pulmonary disease (excluding asthma), renal and liver diseases, cancer, immunosuppression, diabetes, immunosuppression, previous psychiatric illness and dementia. **HFNC**: high-flow nasal cannula oxygen, **CPAP**: Continuous Positive Airway Pressure, **NIV**: Non-invasive Ventilation, **VMK**: Venturi Mask Oxygen. * 4 weeks after hospital demission.
